# Burden‐of‐illness vaccine efficacy

**DOI:** 10.1002/pst.2020

**Published:** 2020-03-27

**Authors:** Andrea Callegaro, Desmond Curran, Sean Matthews

**Affiliations:** ^1^ GSK Vaccines, Rue de l'Institut Rixensart Belgium; ^2^ GSK Vaccines, Avenue Fleming Wavre Belgium

**Keywords:** burden of illness, clinical trials, herpes zoster, vaccine efficacy

## Abstract

In recent years, many vaccines have been developed for the prevention of a variety of diseases. Although the primary objective of vaccination is to prevent disease, vaccination can also reduce the severity of disease in those individuals who develop breakthrough disease. Observations of apparent mitigation of breakthrough disease in vaccine recipients have been reported for a number of vaccine‐preventable diseases such as Herpes Zoster, Influenza, Rotavirus, and Pertussis. The burden‐of‐illness (BOI) score was developed to incorporate the incidence of disease as well as the severity and duration of disease. A severity‐of‐illness score *S* > 0 is assigned to individuals who develop disease and a score of 0 is assigned to uninfected individuals. In this article, we derive the vaccine efficacy statistic (which is the standard statistic for presenting efficacy outcomes in vaccine clinical trials) based on BOI scores, and we extend the method to adjust for baseline covariates. Also, we illustrate it with data from a clinical trial in which the efficacy of a Herpes Zoster vaccine was evaluated.

## 1 INTRODUCTION

One of the main objectives of vaccine efficacy (VE) trials is to compare the proportion of individuals who are infected between vaccinated and unvaccinated groups. However, a vaccine may affect the incidence, duration and severity of a disease. In order to consider incidence, duration, and severity, Chang et al^1^ proposed a simple approach. After assigning a score *S* equal to 0 for uninfected individuals and some postinfection outcome *X* > 0 for infected individuals, they tested the equality of *S* between groups using an adapted *t* test, with a specific variance taking into account the semicontinuous nature of *S*. This method, called burden‐of‐illness (BOI) test, is attractive for two main reasons; first, it is simple and second it is consistent with the intent‐to‐treat (ITT) principle in clinical trials.

Other statistical methods have been proposed in the literature. Follmann et al^2^ proposed a BOI‐like test called Chop‐Lump (CH‐L), which essentially compares the mean of *S* by excluding most of the 0's. This approach is more powerful than BOI in case of no VE. Tu et al^3^ proposed a parametric approach, modeling the *S*'s with a mixture of point mass at zero and a log‐normal distribution. Lachenbruch^4^, ^5^ proposed methods to combine the separate tests for the two endpoints. Mehrotra et al^6^ adjusted the Fisher's method (FCM) for postrandomization selection bias using the potential outcomes framework for causal inference.^7^ They showed that the Fisher's combination test performs best overall, even after adjusting the test for selection bias. Callegaro et al^8^ proposed a permutation‐based Fisher's combination test adjusted for selection bias. Other methods based on causal inferences have also been proposed.^9^, ^10^, ^11^

The VE is the standard statistic in vaccine development studies. For this reason, in this article, we derive a VE measure based on the BOI scores (VE_BOI_). VE_BOI_ is defined as the relative reduction in the BOI score in the vaccinated group compared to the unvaccinated or control group and is calculated as 1 minus the relative risk (RR; the BOI score in the vaccinated group divided by the BOI score in the placebo group). VE_BOI_ is a simple, useful, and interpretable statistic in vaccine development and was chosen as a primary endpoint in the Shingles Prevention Study, which evaluated the VE and safety of a live zoster vaccine.^12^ Furthermore, VE_BOI_ and the following statistic (which is a function of VE_BOI_)

$${\mathrm{VE}}_{\text{onTOP}}=\left({\mathrm{VE}}_{\mathrm{BOI}}‐\mathrm{VE}\right)/\left(1‐\mathrm{VE}\right).$$

have been used to support claims to regulatory agencies.^13^

We illustrate our methods using data from a Herpes Zoster (HZ) clinical trial^14^ that evaluated the VE of adjuvanted Recombinant Zoster Vaccine (RZV) in the prevention of HZ in autologous hematopoietic stem cell transplantation (HSCT) recipients 18 years of age (YOA) and older.

## 2 STATISTICAL METHODS

Let us suppose that *N*_*V*_ volunteers are randomized to receive a vaccine, *N*_*C*_ are randomized to placebo (or control), and that infections are recorded along with a postinfection outcome, *X*. Chang et al^1^ defined the BOI scores *S* as 0 for the uninfected patients and *X* for the infected patients. Chang et al proposed to test the null hypothesis that the distribution of *S* is the same in the two groups using the following *t* test like statistic,

$$t_{\mathrm{BOI}}=\frac{{\bar{S}}_{C}-{\bar{S}}_{V}}{\sqrt{{\mathrm{Var}}_{0}\left({\bar{S}}_{C}-{\bar{S}}_{V}\right)}},$$

where ${\bar{S}}_{j}=1/N_{j}\sum_{i=1}^{N_{j}}S_{i,j}$, for treatment group *j* = *V*, *C*, and *V ar*_0_ is the variance computed under the null hypothesis (score test).

In this article, we consider instead a VE_BOI_ statistic

$${\mathrm{VE}}_{\mathrm{BOI}}=1-\frac{{\bar{S}}_{V}}{{\bar{S}}_{C}},$$

which is a useful and interpretable statistic in vaccine development.

Two different designs are considered: (a) the fixed time design, where the trial is stopped after an fixed duration of follow‐up, and (b) the fixed event design, where the trial is stopped when *n* events are observed. Results of fixed event designs are provided in Appendix A. In case of fixed time design $E\left({\bar{S}}_{j}\right)=p_{j}\mu_{j}$, *j* = *V*, *C*, where *p*_*j*_ is the probability of infection and *μ*_*j*_ is the expectation of *X* of those infected in treatment group *j*. The variance is $\mathrm{Var}\left({\bar{S}}_{j}\right)=p_{j}\left(\sigma_{j}^{2}+\left(1-p_{j}\right)\mu_{j}^{2}\right)/N_{j}$, where $\sigma_{j}^{2}$ is the variance of *X* of those infected in treatment group *j*. Using the Delta method, it follows that

$$\mathrm{Var}\left(\frac{{\bar{S}}_{V}}{{\bar{S}}_{C}}\right)={\left(\frac{p_{V}\mu_{V}}{p_{C}\mu_{C}}\right)}^{2}\left(\frac{p_{V}\left(\sigma_{V}^{2}+\left(1-p_{V}\right)\mu_{V}^{2}\right)}{N_{V}{\left(p_{V}\mu_{V}\right)}^{2}}+\frac{p_{C}\left(\sigma_{C}^{2}+\left(1-p_{C}\right)\mu_{C}^{2}\right)}{N_{C}{\left(p_{C}\mu_{C}\right)}^{2}}\right).$$

### 2.1 Other statistical methods

In this section, we describe in more detail some published approaches and compare these methods via a simulation excercise. We denote by (*prop*) the test which compares the infection rates between the two groups

$$Z_{\text{prop}}=\frac{{\hat{p}}_{C}-{\hat{p}}_{V}}{\sqrt{2\bar{p}\left(1-\bar{p}\right)/M}};$$

and by (*inf*) the *t* test in infected individuals only,

(1) $$Z_{\inf}=\frac{\sum_{i=1}^{n_{C}}X_{C_{i}}/n_{C}-\sum_{i=1}^{n_{V}}X_{V_{i}}/n_{V}}{\sqrt{s_{C}^{2}/n_{C}+s_{V}^{2}/n_{V}}}$$

where *n*_*C*_ and *n*_*V*_ are the number of infected individuals in each group and *X*_*Gi*_ is the *i*th measurement in group *G*. Note that *inf* test can be affected by selection bias because it is based on infected individuals only. The two statistics described above can be combined using the Fisher's test (*FCM*).^6^

(2) $$P‐{\text{value}}_{\mathrm{FCM}}=P\left(\chi_{4}^{2}>-4\log\sqrt{P‐{\text{value}}_{\text{prop}}\times P‐{\text{value}}_{\inf}}\right)$$

whereby *P*‐value1{1}_*inf*_ and *P*‐value_*prop*_ are the one‐sided *P*‐values of *Z*_*inf*_ and *Z*_*prop*_, respectively, and $\chi_{4}^{2}$ represents a random variable with chi‐square distribution and 4° of freedom. Since *FCM* is a function of *inf*, it does not assess a causal effect of vaccine. Mehrotra et al^6^ adjusted *FCM* for postrandomization selection bias using the potential outcomes framework for causal inference. In a similar spirit, Callegaro et al^8^ proposed a permutation‐based FCM test adjusted for selection bias.

Finally, we describe in more details the Chop‐Lump test.^2^ To test the equality of the distribution of *S* between the two treatment groups, all zero observations are removed from the treatment group with fewer zeros and an equal proportion of zeros are removed from the other treatment group. This leaves one group with no zeros at all. The distribution under the null hypothesis is obtained by permutation. The Chop‐Lump statistic is a *t* test similar to the BOI test^1^ calculated on the BOI scores *S*′ on the right of the chopping point,

(3) $$Z_{\mathrm{CH}-\mathrm{LT}}=\frac{\sum_{i=\left(N-l\right)+1}^{N}S_{C_{\left(i\right)}}/l-\sum_{i=\left(N-l\right)+1}^{N}S_{V_{\left(i\right)}}/l}{\sqrt{2s_{l}^{2}/l}}$$

where $s_{l}^{2}$ is the pooled sample variance based on the *l* largest *S*'s in each group, and *l* = max(*n*_*C*_, *n*_*V*_). We considered the rank version of this test (*CH* − *LW*) because it is expected to be more powerful.^2^

## 3 POWER AND SAMPLE SIZE OF VE_BOI_

The asymptotic power under local alternatives is given by

$$\text{Power}=\Phi \left(E\left(Z\right)-z_{1-\alpha /2}\right),$$

where $Z=\log\left(\mathrm{RR}\right)/\sqrt{\mathrm{Var}\left(\log\left(\mathrm{RR}\right)\right)}$ and *RR* = (*p*_*V*_ *μ*_*V*_)/(*p*_*C*_ *μ*_*C*_). If we denote *k*, the randomization ratio (*N*_*V*_ = *kN*_*C*_), then

$$E\left(Z\right)=\sqrt{N_{C}}\frac{\log\left(p_{V}\mu_{V}\right)-\log\left(p_{C}\mu_{C}\right)}{\sqrt{\frac{p_{V}\left(\sigma_{V}^{2}+\left(1-p_{V}\right)\mu_{V}^{2}\right)}{{\left(p_{V}\mu_{V}\right)}^{2}k}+\frac{p_{C}\left(\sigma_{C}^{2}+\left(1-p_{C}\right)\mu_{C}^{2}\right)}{{\left(p_{C}\mu_{C}\right)}^{2}}}}).$$

The sample size is given by

$$N_{C}={\left(\frac{z_{1-\beta }+z_{1-\alpha /2}}{\log\left(p_{V}\mu_{V}\right)-\log\left(p_{C}\mu_{C}\right)}\right)}^{2}\left[\frac{p_{V}\left(\sigma_{V}^{2}+\left(1-p_{V}\right)\mu_{V}^{2}\right)}{{\left(p_{V}\mu_{V}\right)}^{2}k}+\frac{p_{C}\left(\sigma_{C}^{2}+\left(1-p_{C}\right)\mu_{C}^{2}\right)}{{\left(p_{C}\mu_{C}\right)}^{2}}\right].$$

### 3.1 Intention to treat and principal stratum estimator

VE_BOI_ is consistent with the ITT principle because all randomized participants are explicitly included in the analysis. A principal stratum estimator could be considered as well.^10^ The advantage of the ITT approach is the simplicity. In fact, it is much more challenging to apply a principal stratum estimator because membership in the principal stratum must be inferred, usually imperfectly, from covariates. On the other hand, sensitivity analysis based on a principal stratum estimator can be useful to better understand these complex data.

## 4 VE_BOI_ ADJUSTED FOR BASELINE COVARIATES

In this section, we consider the possibility of adjusting the BOI scores for baseline covariates, such as age, sex. In the following, *Z* represents the matrix of baseline covariates. To adjust BOI scores for covariates, we propose a nonlinear regression model where the expectation (*E*) of the BOI score for the *i*th subject is

$$E\left(S_{i}\right)=\exp\left(\alpha +G_{i}\beta_{G}+Z_{i}\beta_{Z}\right),$$

where *G*_*i*_ is the vaccination group (1 = vaccinated; 0 = control). The VE adjusted for covariates is given by

$${\mathrm{VE}}_{\mathrm{BOI}}\left(Z\right)=1-\frac{E\left(S|G=1\right)}{E\left(S|G=0\right)}=1-\exp\left(\beta_{G}\right).$$

this model cannot be fitted using a simple linear regression on the log‐transformed scores, because of the zeros in the scores. In this article, we propose to use a quasi‐Poisson model for the following reasons: (a) the overdispersion parameter allows proper estimation of the variance even in presence of many zeros; (b) the model can be implemented in standard software (such as *R* [glm], or SAS [GLIMMIX]) that does not require integer *S* values. To adjust for the exposure, we included the log of the follow‐up time as an offset. SAS code to fit this model is provided in Appendix B.

## 5 CASE STUDY

In this section, we illustrate our method using data from the GSK HZ clinical trial (NCT01610414) that evaluated the VE of RZV vaccine in the prevention of HZ in autologous HSCT recipients 18 YOA and older.

### 5.1 Description of study

The study was a phase III, observer‐blind randomized, placebo‐controlled, multicenter, multicountry study with two parallel groups to evaluate the VE of RZV vaccine in the prevention of HZ in autologous HSCT recipients 18 YOA and older. Eligible subjects were randomized to RZV or placebo according to a 1:1 ratio. The primary endpoint of this study was to demonstrate the VE of RZV to reduce the number of HZ episodes. However, a secondary objective was to demonstrate a reduction in the severity of pain (including pain triggered by air blowing on the skin, by clothing rubbing against the skin or by hot or cold temperatures) associated with HZ for those subjects experiencing a HZ episode. Pain and other types of discomfort (eg, allodynia and intense pruritus) can have a substantial adverse impact on the functional status and quality of life of affected individuals; therefore, relief of acute and chronic HZ pain and discomfort is an important goal.

### 5.2 Zoster Brief Pain Inventory

The zoster brief pain inventory (ZBPI) was used to quantify HZ pain and discomfort, and was adapted from the Brief Pain Inventory to make it a HZ‐specific measure of pain severity that captures pain and discomfort caused by HZ.^15^ It uses an 11‐point Likert scale (0‐10) to rate HZ pain and discomfort for four dimensions (worst, least, average during the past 24 hours and now) and HZ pain and discomfort‐related interference with seven functional status and ZBPI activities of daily living (ADL) items: general activity, mood, walking ability, work, relations with others, sleep, and enjoyment of life. The seven questions included in the functional status and ADL are summarized into a single score by taking the mean of the seven items.

### 5.3 ZBPI burden‐of‐illness/interference

All subjects with a HZ episode were required to complete the ZBPI questionnaire at the onset of the suspected HZ episode (identified by the presence of rash or pain) on a daily basis from onset of the HZ episode (day HZ‐0) to day HZ‐28, and then weekly onward until a 4‐week pain‐free period was documented. If pain reappeared in the same area after a 4‐week pain‐free period and was not accompanied by a new HZ rash, it was assigned to the previous HZ‐episode.

The HZ BOI scores were calculated from the ZBPI worst pain scores and the HZ burden‐of‐interference score were calculated from the ADL score, over the 182 days from the first day of HZ rash (day HZ‐0) using area under the curve (AUC) methods. The scores were defined as 0 for participants who did not develop an evaluable case of HZ during the study.

The calculation of AUC was based on the trapezoidal rule.^16^

$${\mathrm{AUC}}_{0-m}=\sum_{k=0}^{m-1}\left(t_{k+1}-t_{k}\right)\frac{\left(Y_{k}+Y_{k+1}\right)}{2},$$

where *m* is the number of ZBPI assessments between days 0 and 182, *Y*_*k*_ is the score at timepoint *k* and *t*_*k*_ is the number of days relative to day 0 at timepoint *k*.

### 5.4 Results

#### 5.4.1 Real data analysis

A total of 1721 subjects were included in the analysis cohort. A total of 870 were vaccinated with RZV and 851 subjects were included in the placebo group. About 49 subjects (5.6*%*) in the vaccinated group and 135 subjects (15.9*%*) in the control group developed a case of Herpes Zoster. Of the 184 subjects who developed HZ, 5 did not have a ZBPI score (three in the vaccinated group and two in the placebo group) and were thus removed from the analysis. The distribution of worst ZBPI pain scores is as follows, using the notation in section 2, with *V* = RZV and *C* = Placebo.

$${\bar{S}}_{j}=\frac{\sum_{i=1}^{N_{j}}S_{i,j}}{N_{j}},$$

${\bar{\alpha }}_{j}$ is the mean follow up time (years) in vaccination group *j* = *V*, *C*

$${\bar{S}}_{V}=5.57,{\bar{S}}_{C}=28.70,{\bar{\alpha }}_{V}=1.88,{\bar{\alpha }}_{C}=1.70,$$

$${\mathrm{VE}}_{\mathrm{BOI}}=1-\frac{{\bar{S}}_{V}/\;{\bar{\alpha }}_{V}}{{\bar{S}}_{C}/\;{\bar{\alpha }}_{C}}=0.825,$$

$$\mathrm{Var}\left({\mathrm{VE}}_{\mathrm{BOI}}\right)=\frac{{\bar{\alpha }}_{C}^{2}}{{\bar{\alpha }}_{V}^{2}}\mathrm{Var}\left(\frac{{\bar{S}}_{V}}{{\bar{S}}_{C}}\right)=0.0021.$$

Hence, the 95% confidence interval (CI) of the VE based on the normal distribution is (0.736, 0.914). The 95% CI for the Burden‐of‐Interference is calculated similarly. The VE was also calculated separately for the baseline age categories (18‐49, ≥50 YOA). The results for BOI are presented in Table 1. The VE for Burden‐of‐Interference are presented in Table 2.

**TABLE 1:** Vaccine efficacy of burden‐of‐illness score

Age category	*N*_*V*_ ^a^	*n*_*V*_	${\bar{S}}_{V}$	${\bar{\alpha }}_{V}$	*N*_*C*_	*n*_*C*_	${\bar{S}}_{C}$	${\bar{\alpha }}_{C}$	VE_BOI_ (95% CI)
18‐49 YOA	213	9	3.779	1.98	212	29	20.769	1.80	0.834 (0.634, 1.000)
≥50 YOA	654	37	6.155	1.85	637	104	31.348	1.66	0.824 (0.725, 0.923)
Overall	867	46	5.572	1.88	849	133	28.706	1.70	0.825 (0.736, 0.914)

a *N*_*j*_ = total number in cohort *j*, *n*_*j*_ = total number with disease in cohort *j*, ${\bar{S}}_{j}$ = mean burden score, ${\bar{\alpha }}_{j}$ = mean follow up time (years).

**TABLE 2:** Vaccine efficacy of burden‐of‐interference score

Age category	*N*_*V*_ ^a^	*n*_*V*_	${\bar{S}}_{V}$	${\bar{\alpha }}_{V}$	*N*_*C*_	*n*_*C*_	${\bar{S}}_{C}$	${\bar{\alpha }}_{C}$	VE_BOI_ (95% CI)
18‐49 YOA	213	9	3.368	1.98	212	29	14.994	1.80	0.796 (0.513, 1.000)
≥50 YOA	654	37	3.908	1.85	637	104	21.356	1.66	0.836 (0.739, 0.933)
Overall	867	46	3.778	1.88	849	133	19.767	1.70	0.828 (0.733, 0.923)

Abbreviation: YOA, years of age.

a *N*_*j*_ = total number in cohort *j*, *n*_*j*_ = total number with disease in cohort *j*, ${\bar{S}}_{j}$ = mean burden score, ${\bar{\alpha }}_{j}$ = mean follow up time (years).

#### 5.4.2 BOI Vaccine efficacy adjusting for covariates

The VE for both the BOI and Interference were also calculated adjusting for baseline covariates, using a regression model as described in section 4 (post hoc analysis). Age category at baseline (18‐49, ≥50 YOA) was included as a factor and the log of follow up time was included as an offset. Table 3 shows the results of the burden of illness regression. Age category at baseline was not significant.

**TABLE 3:** Burden‐of‐illness: quasi Poisson regression with follow‐up time as offset

Parameter	Estimate	Std. error	*t* Value	*P* value
Intercept	1.3791	0.2066	6.68	<.0001
Vaccination group	−1.7072	0.3500	−4.88	<.0001
Age category (18‐49 YOA *v* ≥ 50 YOA)	−0.4726	0.3398	−1.39	.1644

Abbreviation: YOA, years of age.

Using the notation from section 4:

$${\mathrm{VE}}_{\mathrm{BOI}}\left(\mathrm{Age}\right)=1-\exp\left(-1.7072\right)=0.819,$$

with 95*%* CI of (0.640, 0.909).

Table 4 shows the results of the burden of interference regression. Age category at baseline was not a significant factor.

**TABLE 4:** Burden‐of‐interference: quasi Poisson regression with follow‐up time as offset

Parameter	Estimate	Std. error	*t* Value	*P* value
Intercept	1.0298	0.2246	4.59	<.0001
Vaccination Group	−1.7239	0.3896	−4.42	<.0001
Age category (18‐49 YOA *v* ≥ 50 YOA)	−0.3673	0.3639	−1.01	.3129

Abbreviation: YOA, years of age.

The overall VE_BOI_ estimate is:

$${\mathrm{VE}}_{\mathrm{BOI}}\left(\mathrm{Age}\right)=1-\exp\left(-1.7239\right)=0.822,$$

with 95% CI of (0.617, 0.917).

## 6 SIMULATION RESULTS

In order to compare the performance of the proposed method with existing approaches, we simulated Zoster Vaccine Trials similar to the real data described above. The number of infections in the placebo group was generated according to a binomial distribution with *N*_*C*_ = *N*_*V*_ = 858 and *p*_*C*_ = 0.15. Different values of VE were considered (VE = [0%, 15%, 30%]), with *p*_*V*_ = *p*_*C*_(1 − *VE*). The log pain score for infected individuals were generated according to a normal distribution with variance *σ* ^2^ = 1.5^2^ and mean *μ*_*C*_ = (4.5, 2.25) or *μ*_*V*_ = (*μ*_*C*_ − Δ) for patients in the placebo or vaccine group, respectively. For each scenario, 10 000 clinical trials were simulated.

We compared the proposed approach (VE_BOI_) with the following approaches: the BOI *t* test^1^ (BOI), the test comparing only infection rates (*prop*), the *t* test comparing postinfection outcomes in infected individuals (*inf*), the Fisher's test (*FCM*) combining *prop* and *inf* ^6^ and the rank version of the Chop‐Lump test (*CH* − *LW*).^2^

Table 5 shows simulation results based on our real Zoster data. As expected, the power of VE_BOI_ increases as VE or Δ increases. We focus now on the comparisons between the proposed method and the BOI and Chop‐Lump tests. First of all we notice that the power of VE_BOI_ is similar to the power of BOI (only slightly better). This is not surprising, the advantage of VE_BOI_ over BOI being the interpretability of the estimated effect. When Δ = 0 (and presumably for very small values) these three tests have similar power, and no method performs very well. At larger Δ values, VE_BOI_ performs better, but generally has lower power than the Chop‐Lump test, unless the VE is “large” (VE = 30%). This is due to the ratio *μ*_*C*_/*σ* = 3. In fact, asymptotic and simulation results of Follmann et al^2^ shows that the power of BOI (and VE_BOI_ as well) decreases with larger values of *μ*_*C*_/*σ*. The last row of Table 5 shows the hypothetical case when *μ*_*C*_ is 2.25 instead of 4.5 so the ratio is much smaller (*μ*_*C*_/*σ* = 1.5). As expected,^2^ in this case VE_BOI_ (and BOI) is more powerful, even more powerful than *CH* − *LW*.

**TABLE 5:** Power for different tests for HZ Pain and infection in a Phase III zoster vaccine trial

VE (%)	*p*_*C*_	*p*_*V*_	Δ	*μ*_*C*_	*μ*_*V*_	*V E*_BOI_	BOI	CH‐LW	FCM	*prop*	*inf*
0	0.15	0.1500	0.0	4.50	4.50	0.024	0.023	0.024	0.024	0.024	0.026
15	0.15	0.1275	0.0	4.50	4.50	0.239	0.235	0.202	0.186	0.267	0.025
30	0.15	0.1050	0.0	4.50	4.50	0.757	0.752	0.680	0.683	0.801	0.023
0	0.15	0.1500	0.4	4.50	4.10	0.114	0.109	0.206	0.426	0.023	0.556
15	0.15	0.1275	0.4	4.50	4.10	0.502	0.495	0.590	0.650	0.268	0.529
30	0.15	0.1050	0.4	4.50	4.10	0.910	0.906	0.921	0.910	0.802	0.497
0	0.15	0.1500	0.8	4.50	3.70	0.351	0.344	0.598	0.967	0.026	0.988
15	0.15	0.1275	0.8	4.50	3.70	0.782	0.775	0.906	0.982	0.268	0.981
30	0.15	0.1050	0.8	4.50	3.70	0.980	0.979	0.992	0.997	0.792	0.970
0	0.15	0.1500	0.8	2.25	1.45	0.714	0.662	0.587	0.964	0.022	0.988

*Note*: A total of 1716 subjects were randomized (1:1 randomization ratio) and had a control infection rate of 15%.

Finally, we compare VE_BOI_ with *FCM* test. With the exception of small Δ or” large” VE (VE = 30%), *FCM* is more powerful than VE_BOI_, however this test is not consistent with the ITT principle because *Z*_*inf*_ is restricted to subjects who are selected based on a postrandomization event. Note that *Z*_*inf*_ (and consequently *FCM*) can lead to the conclusion that a harmful vaccine generating more infections with low pain is beneficial.

## 7 CONCLUSIONS

In vaccine clinical trials, the VE estimate is the standard statistic for presenting efficacy outcomes. In this article, we proposed a VE statistic based on the BOI score.^1^ Even if more advanced methods have been proposed in the literature,^2^, ^3^, ^4^, ^5^, ^6^, ^8^, ^9^, ^10^, ^11^ we believe that this approach has an important role from a practical point of view. First, the statistic is meaningful and interpretable in vaccine clinical trials: it is one minus the BOI score ratio and represents the proportional reduction in BOI score due to the vaccine. Second, because all randomized participants are explicitly included in the analysis, our method is consistent with the intent‐to‐treat principle. A principal stratum estimator could be considered as well^10^ and can be useful to better understand these complex data, but it is more complex because membership in the principal stratum must be inferred, usually imperfectly, from covariates. Third, it is simple to apply because it is a regression method that can be performed using standard software and can easily be extended to involve more complex analyses. A possible extension of the proposed BOI regression method is a multivariate outcome approach, where multiple severity scores (such as pain, vomiting, …) are modeled using multivariate regression approaches, such as mixed‐effects regression models and generalized estimating equation models.

As a motivating example, we analysed HZ data from a vaccine clinical trial. The overall HZ VE for HZ incidence was reported as 68.2%.^17^ The overall VE for HZ‐BOI was estimated to be 82.5%. The following statistic may be used to support claims to regulatory agencies:

$${\mathrm{VE}}_{\text{onTOP}}=\left({\mathrm{VE}}_{\mathrm{BOI}}‐{\mathrm{VE}}_{\mathrm{HZ}}\right)/\left(1‐{\mathrm{VE}}_{\mathrm{HZ}}\right)=45.0\%.$$

However, it should be noted that VE_onTOP_ is conditional on developing disease, that is, it can easily be shown that VE_onTOP_ corresponds to 1 − *μ*_*V*_/*μ*_*C*_. As such, the statistic is subject to selection bias which could lead to the conclusion that a harmful vaccine generating more infections with low pain is beneficial. Consequently the VE_onTOP_ statistic should (a) be adjusted for selection bias, or (b) always be reported in conjunction with the VE_HZ_ and VE_BOI_ statistics to provide a complete overview of results. Further research needs to explore how this statistic can be interpreted and used in future research.

Similarly, the overall VE for reducing the Burden‐of‐Interference of HZ on activities of daily living was estimated to be 82.8%. The results from this analysis would suggest that in addition to preventing HZ, vaccination with RZV also reduces the BOI and Burden‐of‐Interference in subjects who develop HZ. Plausible hypotheses are that memory CD4+ T cells would be capable of mounting a rapid antiviral response upon reactivation of the varicella zoster virus. In some cases, this anamnestic immune response may not be able to prevent a HZ episode but in vaccine recipients with breakthrough disease, the response may be sufficient to more rapidly control the reactivated virus, leading to reduced severity of disease.^18^ Apparent mitigation of breakthrough disease in vaccine recipients have also been reported for a number of other vaccine‐preventable diseases such as influenza, rotavirus, and pertussis.^19^, ^20^, ^21^

We proposed a quasi‐Poisson regression model on the scores to adjust the BOI VE for baseline covariates. The overdispersion parameter allows proper estimation of the variance even in presence of many zeros and the model can be implemented in standard software (such as *R* [glm], or SAS [GLIMMIX]) that does not require integer *S* values. It is straightforward to extend the model, for example by including the interaction between treatment and covariates. In this way, it is possible to test if the VE_BOI_ depends on baseline covariates (testing if the interaction is significant).

Simulation results based on our real data showed that the power of VE_BOI_ is similar to the power of BOI. As expected, the advantage of VE_BOI_ over BOI is not in terms of power but of the interpretability of the estimated effect. In agreement with simulation results of Follmann et al,^2^ Chop‐Lump (rank) test is more powerful than the VE_BOI_ test when there is small VE and large ratio *μ*_*C*_/*σ*. Future research could evaluate the proposed BOI regression using postinfection values which have been transformed (*f*[*S*]) to reduce the ratio $E(f\left(S|C\right)/\sqrt{\mathrm{Var}(f\left(S|C\right)}$).

In summary, the proposed BOI VE is a useful and interpretable measure of efficacy for clinical trials where the vaccine may affect both the incidence and the severity of disease. The adjustment for baseline covariates can further improve the efficiency of the analysis and avoid conditional bias from chance covariate imbalance.

## CONFLICT OF INTEREST

AC and DC are employees of and holds shares in the GSK group of companies. SM is a freelance consultant on behalf of GSK.

## AUTHOR CONTRIBUTIONS

All authors contributed equally to this work.

## Data Availability

Anonymized individual participant data and study documents can be requested for further research from www. clinicalstudydatarequest.com.

## Fixed number of event design

In case of fixed number of events design, it follows that *n*_*C*_ ∼ *B*(*n*, *π*_*C*_) where *π*_*C*_ is the proportion of events in the control group (*π*_*C*_ = *n*_*C*_/*n*). It follows that $E\left({\bar{S}}_{j}|n\right)=\pi_{j}\mu_{j}\times n/N_{j}$ and $\mathrm{Var}\left({\bar{S}}_{j}|n\right)=\pi_{j}\left(\sigma_{j}^{2}+\left(1-\pi_{j}\right)\mu_{j}^{2}\right)n/N_{j}^{2}$. In this case, the two means are not independent (*Cov*(*n*_*C*_, *n*_*V*_|*n*) = −*V ar*(*n*_*C*_|*n*) = *nπ*_*C*_(1 − *π*_*C*_)) and $\mathrm{Cov}\left({\bar{S}}_{C},{\bar{S}}_{V}|n\right)=-n\pi_{V}\pi_{C}\mu_{V}\mu_{C}/\left(N_{V}N_{C}\right)$. Using Delta method, it follows that

$$\mathrm{Var}\left(\frac{{\bar{S}}_{V}}{{\bar{S}}_{C}}|n\right)={\left(\frac{\pi_{V}\mu_{V}/N_{V}}{\pi_{C}\mu_{C}/N_{C}}\right)}^{2}\left(\frac{\pi_{V}\left(\sigma_{V}^{2}+\left(1-\pi_{V}\right)\mu_{V}^{2}\right)}{n{\left(\pi_{V}\mu_{V}\right)}^{2}}+\frac{\pi_{C}\left(\sigma_{C}^{2}+\left(1-\pi_{C}\right)\mu_{C}^{2}\right)}{n{\left(\pi_{C}\mu_{C}\right)}^{2}}+\frac{2}{n}\right).$$

The expectation of the statistic $Z=\log\left(\mathrm{RR}\right)/\sqrt{\mathrm{Var}\left(\log\left(\mathrm{RR}\right)\right)}$ is given by

$$E\left(Z|n\right)=\sqrt{n}\frac{\log\left(\pi_{V}\mu_{V}/k\right)-\log\left(\pi_{C}\mu_{C}\right)}{\sqrt{\frac{\pi_{V}\left(\sigma_{V}^{2}+\left(1-\pi_{V}\right)\mu_{V}^{2}\right)}{{\left(\pi_{V}\mu_{V}\right)}^{2}}+\frac{\pi_{C}\left(\sigma_{C}^{2}+\left(1-\pi_{C}\right)\mu_{C}^{2}\right)}{{\left(\pi_{C}\mu_{C}\right)}^{2}}+2}}),$$

and the sample size is

$$n={\left(\frac{z_{1-\beta }+z_{1-\alpha /2}}{\log\left(\pi_{V}\mu_{V}/k\right)-\log\left(\pi_{C}\mu_{C}\right))}\right)}^{2}\left[\frac{\pi_{V}\left(\sigma_{V}^{2}+\left(1-\pi_{V}\right)\mu_{V}^{2}\right)}{{\left(\pi_{V}\mu_{V}\right)}^{2}}+\frac{\pi_{C}\left(\sigma_{C}^{2}+\left(1-\pi_{C}\right)\mu_{C}^{2}\right)}{{\left(\pi_{C}\mu_{C}\right)}^{2}}+2\right].$$

## SAS code to fit BOI model

We present here SAS code used to fit the BOI model.

PROC GLIMMIX data = data;

MODEL S=G AGE/ OFFSET=logtime link = log;

_variance_ = _mu_;

random _residual_;

RUN;
